# Identification of cuproptosis-realated key genes and pathways in Parkinson’s disease via bioinformatics analysis

**DOI:** 10.1371/journal.pone.0299898

**Published:** 2024-04-16

**Authors:** Jia Song, Jia Li, Xiaochen Pei, Jiajun Chen, Lin Wang

**Affiliations:** Department of Neurology, China-Japan Union Hospital of Jilin University, Changchun, China; University of Science and Technology Liaoning, CHINA

## Abstract

**Introduction:**

Parkinson’s disease (PD) is the second most common worldwide age-related neurodegenerative disorder without effective treatments. Cuproptosis is a newly proposed conception of cell death extensively studied in oncological diseases. Currently, whether cuproptosis contributes to PD remains largely unclear.

**Methods:**

The dataset GSE22491 was studied as the training dataset, and GSE100054 was the validation dataset. According to the expression levels of cuproptosis-related genes (CRGs) and differentially expressed genes (DEGs) between PD patients and normal samples, we obtained the differentially expressed CRGs. The protein-protein interaction (PPI) network was achieved through the Search Tool for the Retrieval of Interacting Genes. Meanwhile, the disease-associated module genes were screened from the weighted gene co-expression network analysis (WGCNA). Afterward, the intersection genes of WGCNA and PPI were obtained and enriched using the Gene Ontology (GO) and Kyoto Encyclopedia of Genes and Genomes (KEGG). Subsequently, the key genes were identified from the datasets. The receiver operating characteristic curves were plotted and a PPI network was constructed, and the PD-related miRNAs and key genes-related miRNAs were intersected and enriched. Finally, the 2 hub genes were verified via qRT-PCR in the cell model of the PD and the control group.

**Results:**

525 DEGs in the dataset GSE22491 were identified, including 128 upregulated genes and 397 downregulated genes. Based on the PPI network, 41 genes were obtained. Additionally, the dataset was integrated into 34 modules by WGCNA. 36 intersection genes found from WGCNA and PPI were significantly abundant in 7 pathways. The expression levels of the genes were validated, and 2 key genes were obtained, namely peptidase inhibitor 3 (PI3) and neuroserpin family I member 1 (SERPINI1). PD-related miRNAs and key genes-related miRNAs were intersected into 29 miRNAs including hsa-miR-30c-2-3p. At last, the qRT-PCR results of 2 hub genes showed that the expressions of mRNA were up-regulated in PD.

**Conclusion:**

Taken together, this study demonstrates the coordination of cuproptosis in PD. The key genes and miRNAs offer novel perspectives in the pathogenesis and molecular targeting treatment for PD.

## 1. Introduction

Parkinson’s disease (PD) is the second most common neurodegenerative disease with a global prevalence of more than 6 million individuals [1], second only to Alzheimer’s disease (AD). To date, there are no agents shown to have unequivocal evidence of disease-modifying effects in PD, which is hazardous to human health [2]. Common clinical manifestations include motor symptoms such as tremor, myotonia, bradykinesia, and postural balance disorders, as well as non-motor symptoms such as sensory and autonomic symptoms [3]. Pathologically, PD is characterized by a loss of dopaminergic neurons in the substantia nigra and the presence of Lewy bodies composed of abnormal accumulation of α-synuclein in the midbrain [4]. However, the pathogenesis of PD is not conclusive and the metal ion dyshomeostasis [5], oxidative stress [6], mitochondrial dysfunction [7], and chronic inflammation [8] may be involved. In addition, genetic factors play an important role in the onset and progression of PD. Generally, rare DNA variants are typically associated with monogenic or familial PD; and more common, smaller effect variants are usually identified in apparently sporadic PD [9]. Therefore, identifying related genes and exploring the possible pathways that may be involved in PD pathogenesis could provide new ideas for the prediction and intervention of the disease.

Copper is important for various neuronal functions such as interacting with synaptic proteins and neurotransmitter receptors in synapses [10]. In vitro studies have shown that the presence of copper in millimolar concentrations causes the formation of partially folded amyloidogenic conformations with a propensity for aggregation [11]. Mechanically, copper dysfunction is associated with different toxic effects, mainly represented by oxidative stress [12]. Therefore, careful homeostatic control of copper levels plays an important role in PD pathology, leading to the development of novel therapeutic approaches based on restoring copper homeostasis [11]. Currently, experimental evidence has proven the inhibition of α-synuclein oligomer-mediated reactive oxygen species by copper chelators [13]. Cuproptosis is a novel form of cell death newly proposed [14]. It occurs via the direct binding of copper to lipoylated components of the tricarboxylic acid (TCA) cycle in the mitochondria, leading to proteotoxic stress and ultimately cell death. As is well acknowledged, the key role of mitochondrial dysfunction has been demonstrated in PD, and several potential therapeutic avenues targeting to promote the clearance of old or damaged mitochondria have been consistently developed [15]. A recent study showed that isogenic human induced pluripotent stem cells with PARK2 knockout displayed abnormal TCA cycle activity, perturbed mitochondrial ultrastructure, and increased oxidative stress [16]. Therefore, it would be reasonable to infer that cuproptosis is closely associated with PD. However, the potential regulatory mechanisms remain unknown and need further exploration. What’s more, the identification of key genes and miRNAs in PD might assist to explain the correlation from a genetic perspective.

Predicting key differentially expressed genes (DEGs) for disease by comprehensive biochemical computational methods is a prevalent approach that efficiently and rapidly offers molecular insights into the disease utilizing a variety of resources of sequencing results in a high-throughput manner. Currently, there have been some studies predicting the cuproptosis-related biomarkers of the neurodegenerative diseases based on bioinformatics tools [17–20]. Lai et al. [17] combined the weighted gene co-expression network analysis (WGCNA) algorithm with several machine models such as random forest model to explore the cuproptosis-related molecular clusters and construct a prediction model for AD. As for PD, few studies [18–20] aimed to discover the DEGs about cuproptosis by means of different computational biological methods. A recent bioinformatics analysis [20] found that the cuprotosis-related key genes intervened in the progression of PD through the integration of DEGs, WGCNA-related significant module genes (with the soft threshold of 10) and immune cell infiltration, resulting in three key genes different from our study, which might be attributed to the different selection of parameters. However, all the genes obtained from the comprehensive bioinformatics analyses would be of great importance for the exploration of molecular perspectives on disease.

**Fig 1:**
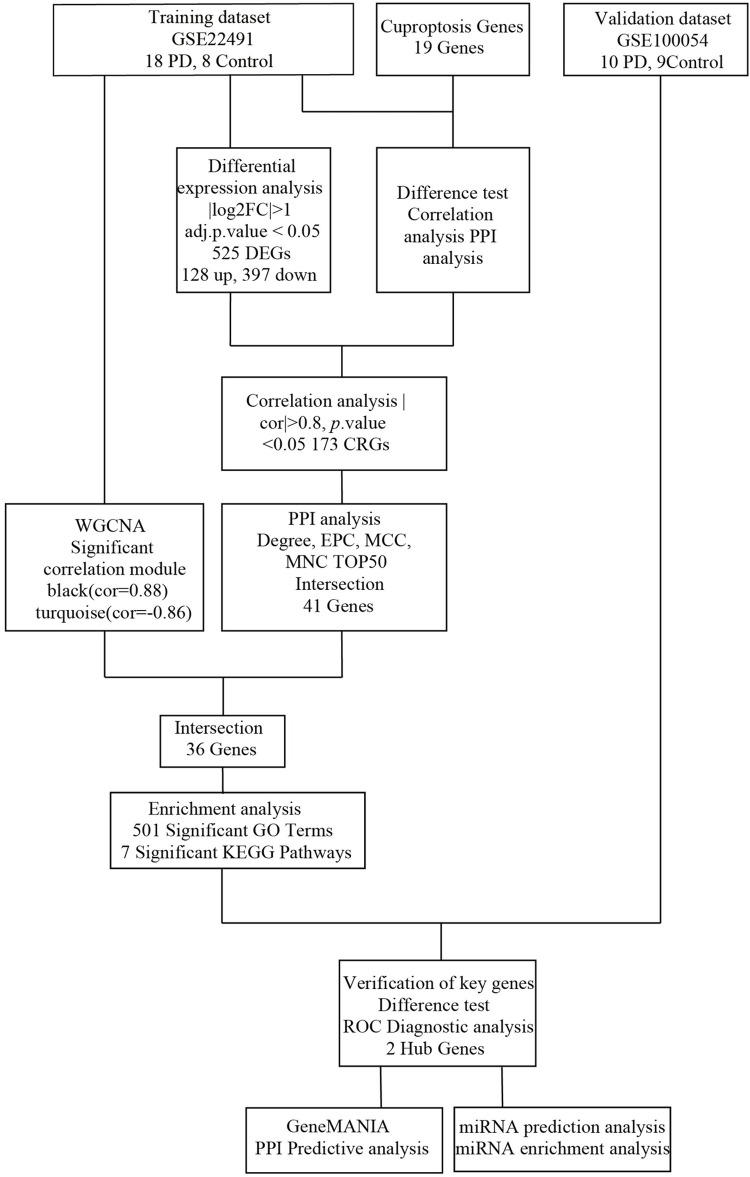
The flow chart of the bioinformatics analysis.

In this research, we acquired 10 PD and 8 normal samples in the GSE22491 database as the training dataset, and 10 PD and 9 normal samples in the GSE100054 as the validation dataset. Based on the expression levels of cuproptosis-related genes (CRGs) and DEGs between PD and normal samples, a correlation analysis was performed to observe the differentially expressed CRGs. A protein-protein interaction (PPI) network was performed using the Search Tool for the Retrieval of Interacting Genes (STRING). Meanwhile, we screened for disease-associated module genes by the WGCNA and took the intersection of significantly related modular genes in the results of WGCNA and the genes from PPI, which were enriched by the Kyoto Encyclopedia of Genes and Genomes (KEGG) and Gene Ontology (GO) in succession. Then the expression levels of the intersection genes were validated in the training and validation datasets to identify two key genes, peptidase inhibitor 3 (PI3) and neuroserpin family I member 1 (SERPINI1). Next, we plotted the receiver operating characteristic (ROC) curves in all datasets and constructed a PPI network. Finally, 29 key miRNAs were screened by taking the intersection of PD-related miRNAs and key genes-related miRNAs and enriched by the KEGG analysis. A detailed flow chart of the bioinformatics analysis was exhibited (Fig 1). Finally, the mRNA expressions of PI3 and SERPINI1 were verified from the qRT-PCR results. Through the above research, it is concluded that cuproptosis has effects on PD pathogenesis. Moreover, these genes and factors may be potential targets for therapeutic drugs.

## 2. Materials and methods

### 2.1 Data acquisition

The datasets used in this analysis were obtained from the NCBI GEO database [21] (https://www.ncbi.nlm.nih.gov/). We searched for PD-related datasets using "Parkinson’s disease", obtained the pre-processed, normalized and log2-transformed probe expression matrix, followed by downloading the platform annotation file. The probes that did not match the Gene symbol were removed. For different probes mapping to the same gene, the mean value of different probes was taken as the final expression value of the gene. GSE22491 was selected as the training dataset with 18 samples, including 10 PD and 8 normal samples, and the sequencing platform was GPL6480 (Agilent-014850 Whole Human Genome Microarray 4x44K G4112F (Probe Name version)). In addition, GSE100054 was selected as the validation dataset. 19 samples, including 10 PD and 9 normal samples, were sequenced on GPL23126 ([Clariom_D_Human] Affymetrix Human Clariom D Assay [transcript (gene) version]). Data were freely available online, and our study did not involve any experiments in the lab performed by any of the authors.

### 2.2 Differential analysis of CRGs

19 CRGs were obtained from the relevant literature [22]. The expression levels of the genes were extracted for differential analysis from the training dataset between the PD and normal samples. The rank sum test was used for differential analysis. It is the method of hypothesis testing commonly used between two groups of samples to test whether the difference of a certain indicator is significant. Then a correlation analysis was performed among the genes. Correlation analysis was performed using the pearson algorithm of cor function in R (version 3.6.2). Pearson correlation analysis is a method to analyze the linear correlation between the two variables. The PPI network was analyzed using the STRING [23] (Version: 10.0, http://www.string-db.org/) database, with the input gene set of CRGs and species of homo. A score of 0.4 (medium confidence) was selected as the cutoff criterion, requiring that the interacting protein nodes were included in the above genes.

### 2.3 DEGs and differentially expressed CRGs screening

The R software limma package [24] (Version 3.10.3, http://www.bioconductor.org/packages/2.9/bioc/html/limma.html) was used to perform DEGs analysis in the training dataset. Limma, a type of generalized linear model that fits a linear equation to the expression of each gene, is a common method for screening for the DEGs. False Discovery Rate (FDR)<0.05 and |logFC|>1 were set as the threshold to screen the DEGs.

Correlation analysis of CRGs and DEGs was performed using the pearson algorithm of cor package in R. DEGs whose correlation coefficients with at least one CRG were greater than 0.8 were selected as differentially expressed CRGs, which were used for further screening analysis.

### 2.4 PPI network construction for differentially expressed CRGs

The reciprocal relationships among differentially expressed CRGs encoding proteins were analyzed from the STRING database. The database contains 1380838440 interactions of 9643763 proteins from 2031 species, which could be used for protein interaction analysis. The graph was constructed using the Cytoscape software [25] (version 3.4.0, http://chianti.ucsd.edu/cytoscape-3.4.0/).

In addition, we applied the CytoNCA [26] plug-in (Version 2.1.6, http://apps.cytoscape.org/apps/cytonca) to analyze the nodal topological properties of the network, where Degree, EPC, MCC, and MNC were used as the main attributes. CytoNCA is a cytoscape plugin for centralized analysis and evaluation of biological networks, so that the key nodes could be filtered from the interworking networks. The larger value of each attribute proved the greater role of the gene in the network. The TOP 50 genes were selected under each attribute in turn and the key differentially expressed CRGs were obtained by taking the intersection.

### 2.5 WGCNA screening for disease-related modules

WGCNA is a systematic biological method to characterize the modules of gene association between samples. It could be used to identify the highly synergistic gene modules which could be served as the candidate biomarkers or therapeutic targets based on the introgression and association between the gene set modules and the disease traits. The input genes were analyzed using the R package WGCNA [27] (Version 1.61, https://cran.r-project.org/web/packages/WGCNA/) based on the dataset GSE22491 to define modules of highly correlated genes associated with PD. In the WGCNA algorithm, the element in the defined gene co-expression modules was the weighted value of the correlation coefficient, and it was chosen so that the networks were scale-free. The weighted value here was the soft Power. The parameters (min Module Size = 30; merge Cut Height = 0.25) were set to aggregate highly correlated genes into modules based on the clustering and dynamic pruning methods. Finally, the modules that are correlated with the disease with the *P*-value less than 0.05 were selected as disease-related modules by analyzing the correlation between the module and the clinical traits (whether the sample is a diseased or normal sample). The genes in the modules with the most positive and negative correlation were selected as the genes associated with PD.

### 2.6 Intersection genes screening

Disease-related module genes were obtained by WGCNA, and the key differentially expressed CRGs were obtained by the PPI analysis. They were intersected for further study.

### 2.7 Gene enrichment analysis

The enrichment analysis is a statistical method widely used in the bioinformatics analyses, which is mainly applied to identify the biologically significant patterns and functions from a large amount of genetic information. GO analysis is commonly used to test the enrichment of Gene Ontology entries in a collection of genes and predict the common features of the genes in terms of biological process (BP), molecular function (MF) and cellular component (CC), while KEGG is used to analyze the role of genes in metabolic and signaling pathways.

The GO [28] function enrichment analysis and KEGG [29] pathway enrichment analysis were performed using R package clusterProfiler [30]. The GO analysis terms included CC, MF, and BP. *P*<0.05 was considered significant due to the few enriched pathways.

### 2.8 Identification of key genes

The box plots of the screened genes were used to show the expression levels in the training and validation datasets. The rank sum test was used to identify the key genes (*P*<0.05), requiring they were consistent up or down-regulation trend in the datasets.

### 2.9 Diagnostic accuracy assessment of key genes and PPI network construction

The pROC package Version 1.12.1 [31] (https://cran.r-project.org/web/packages/pROC/index.html) in R language was used to plot ROC curves in all datasets to assess the diagnostic accuracy. The Area Under Curve (AUC) was utilized to assess the area under the ROC curve enclosed with the coordinate axis. The PPI analysis of the key genes was performed using the GeneMANIA database (https://genemania.org/) [32]. The database could predict the proteins in terms of co-localization, shared protein structural domains, and the correlation with the signaling pathways, etc.

### 2.10 Key miRNAs screening

HMDD is a hand-collected database to explore the miRNAs associated with the disease. The HMDD V3.0 database [33] (http://www.cuilab.cn/hmdd) was used to retrieve PD -related miRNAs, and miRWalk (http://mirwalk.umm.uni-heidelberg.de/) [34] was used to predict miRNAs for key genes. They were intersected to obtain the key miRNAs, and a gene-miRNA network was constructed.

### 2.11 KEGG pathway enrichment analysis of key miRNAs

The KEGG pathway analysis was performed on the key miRNAs through DIANA-miRPath v3.0 [35] (http://www.microrna.gr/miRPathv3/). DIANA-mirPath is a web server for miRNA pathway analysis, through which the miRNA enrichment could be analyzed. Pathways showing FDR<0.05 were considered significantly enriched.

### 2.12 RT-qPCR confirmation

Reverse transcription qPCR was used to quantify the amount of mRNA measured by the fluorescence quantitative PCR instrument from Beckman, USA. The cell model of PD was constructed with 1 mM MPP+ (sigma) treatment of SH-SY5Y cells. Total RNA from SH-SY5Y cells was extracted with Trizol reagent (leagene, China). The cDNA was generated immediately from 1 μg extracted RNA by HiScript II Q RT SuperMix for qPCR (Vazyme, China). Then quantitative PCR was performed by ChamQ Universal SYBR qPCR Master Mix (Vazyme, China). The relative gene expression was analyzed by means of the 2^–ΔΔCt^ method. The primers were designed to target PI3 and SERPINI1. The sequences are as follows: PI3: Forward: TGTCAAAGGCCGTGTTCCAT, Reverse: GAGCCAGGCTTAGTGGAGAC; SERPINI1: Forward: AGCAATTCACAAGTCCTTCCTAGAG, Reverse: CTTGAGGATACAGCACAGCCATC.

## 3. Results

### 3.1 Differential analysis of CRGs

**Fig 2:**
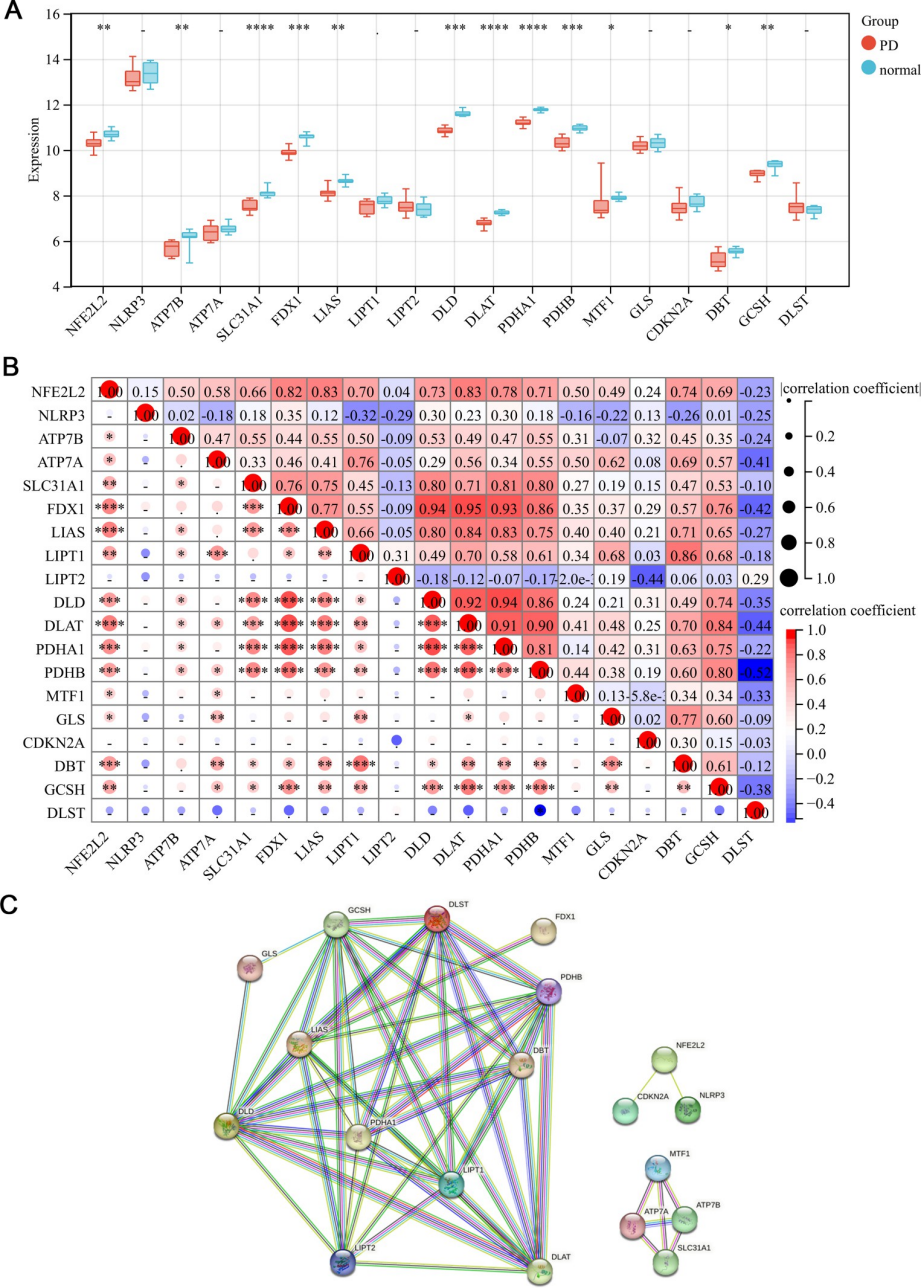
The differential analysis of CRGs. (A) The box plot of CRGs expression levels between PD and normal samples. (B) The heatmap of CRGs. (C) The PPI network of CRGs. *: *P*<0.05; **: *P*<0.01; ***: *P*<0.001; ****: *P*<0.0001; the remaining symbols: not statistically significant (*P*≥0.05).

Based on the expression levels of CRGs (S1 Table) in the training dataset, differential analysis between PD and normal samples was performed and a box plot was drawn (Fig 2A). To explore the association of CRGs, the Pearson’s correlation coefficient was calculated and a heatmap was drawn to present the results (Fig 2B). The PPI network of CRGs was performed based on the STRING database (Fig 2C).

### 3.2 Differentially expressed CRGs screening and PPI network construction

**Fig 3:**
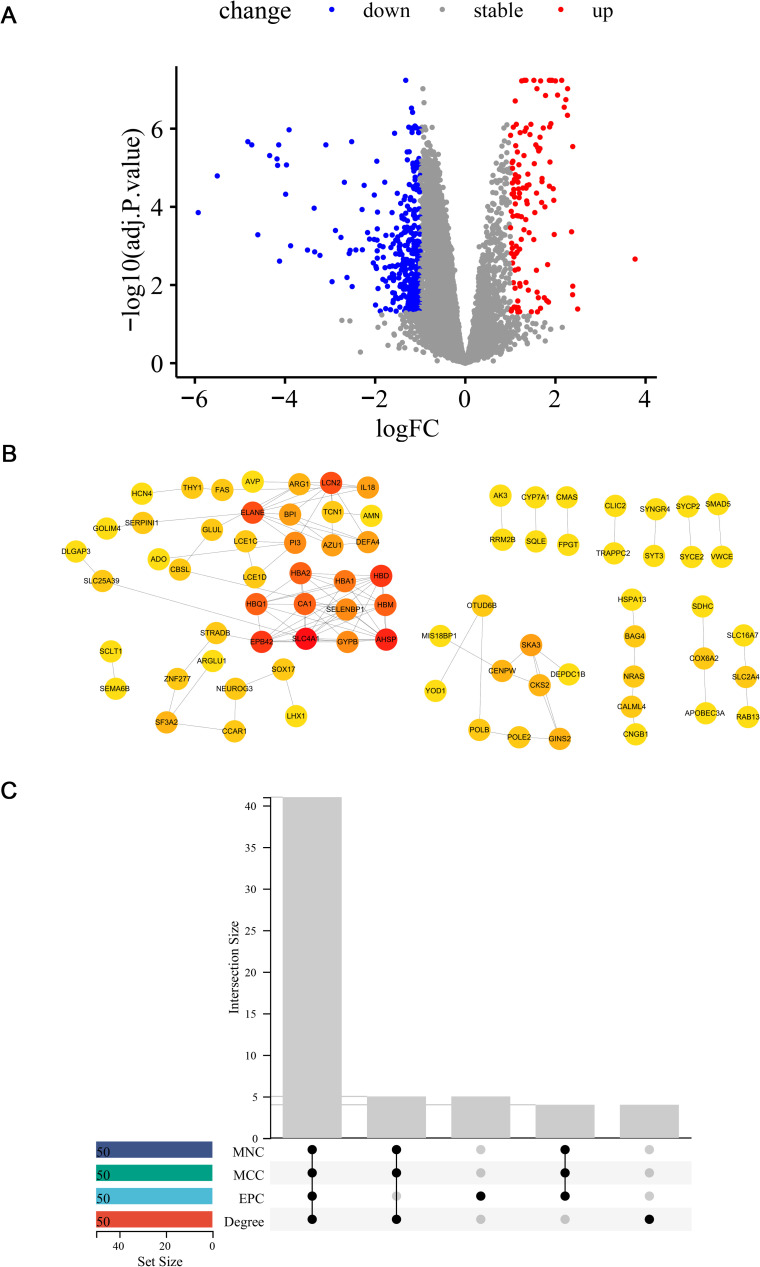
DEGs screening and PPI network construction. (A) The volcano plot of DEGs (red indicated up-regulated genes, blue indicated down-regulated genes, and gray indicated genes with insignificant differences of expression levels). (B) The PPI network construction for differentially CRGs. The gray connecting lines indicated the interactions between the corresponding proteins of genes. Red indicated genes with more connecting lines, and yellow indicated genes with fewer connecting lines. (C) The upset diagram taking the intersection of the TOP 50 genes of each nodal topological property in the PPI network.

128 up-regulated and 397 down-regulated genes were obtained from the training dataset, altogether 525 DEGs. Based on the screened differential genes, a volcano plot was performed (Fig 3A).

Correlation analysis of CRGs and DEGs was performed and 173 differentially CRGs were screened in total. Further, a PPI network was performed (Fig 3B). We obtained a reciprocal network consisting of 79 genes, demonstrating that these genes had close interactions and might play an important role in the disease. The nodal topological properties of the network were analyzed, and the TOP 50 genes of the four attributes were selected to take the intersection (Fig 3C). 41 key differentially expressed CRGs were observed.

### 3.3 WGCNA and intersection genes screening

**Fig 4:**
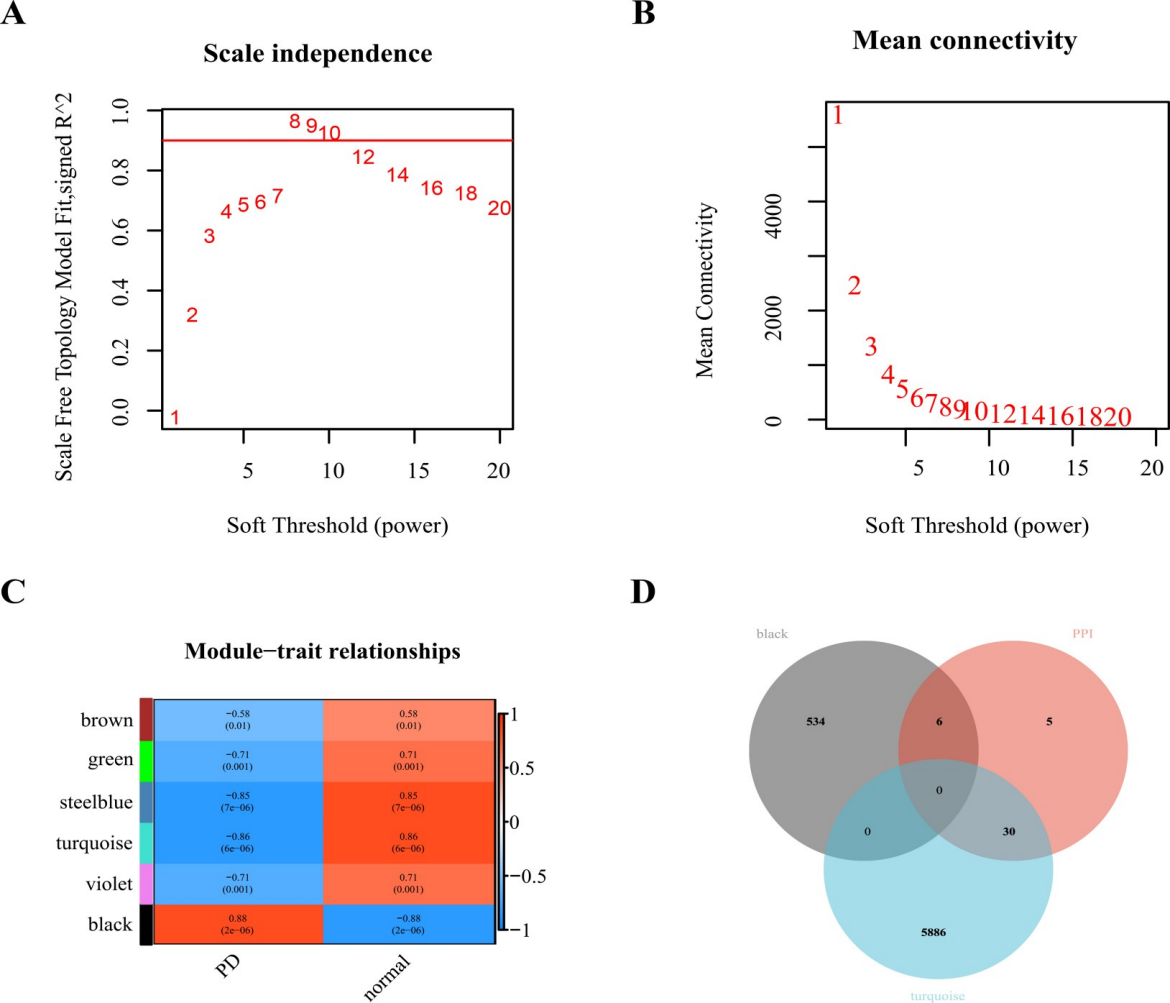
WGCNA and intersection genes screening. (A) The analysis of network topology for various soft-thresholding powers. The x-axis reflected the soft-thresholding power, and the y-axis reflected the scale-free topology model fit index. Here we selected the soft threshold corresponding to the ordinate reaching 0.9 for the first time, which was 8. (B) The x-axis reflected the soft-thresholding power, and the y-axis reflected the mean connectivity (degree). (C) The correlation analysis between gene modules and clinical traits. The upper numbers indicated the correlation coefficients, and the lower bracketed numbers indicated *P* values. (D) The Venn diagram obtained by taking the intersection of PD-related key module genes and PPI key genes.

WGCNA was performed on the training dataset, and the soft threshold was 8 (Fig 4A and 4B). Next, the highly correlated genes were aggregated into modules based on the clustering and dynamic pruning method, and these modules were clustered with the coefficients of dissimilarity of 0.25. Highly correlated genes were aggregated into 34 modules. Further, the correlation between the eigenvector gene of each module and the clinical traits was calculated and the modules with a significant *P*-value was plotted (Fig 4C). The black module (540 genes; correlation coefficient r = 0.88 and *P*.value<0.001) showed the strongest positive correlation with PD, so this module was taken as the key module associated with PD. The turquoise module (5916 genes; correlation coefficient r = -0.86 and *P*.value<0.001) showed the strongest negative correlation with PD, so this module was also considered a PD-related key module.

There were 36 genes (S2 Table) obtained by intersecting the black and turquoise modular genes from WGCNA and the genes from PPI (Fig 4D).

### 3.4 Gene enrichment analysis

**Fig 5:**
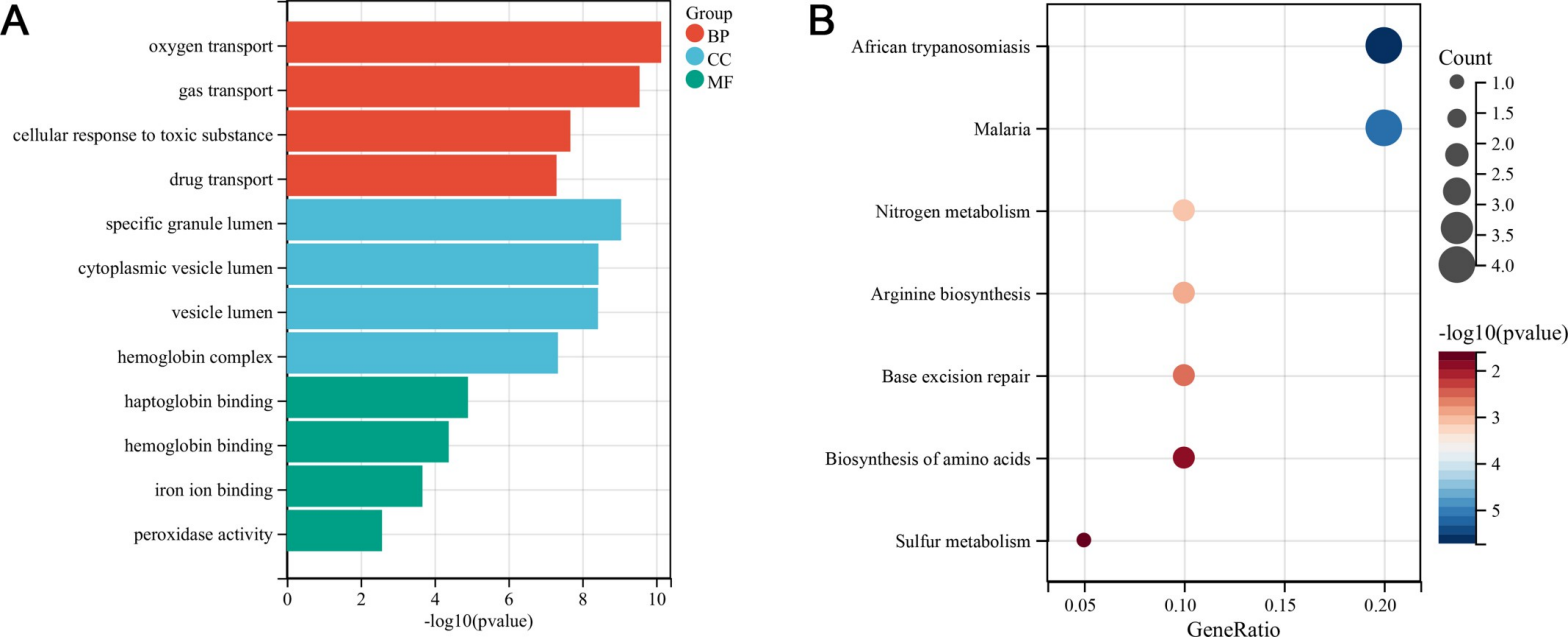
Gene enrichment analysis. (A) The GO term histogram of the intersection genes. The 4 GO terms with the smallest *P* values in each group was displayed. (B) The KEGG pathway bubble graph of the intersection genes.

GO and KEGG were performed on the above-obtained genes. 501 terms were enriched by the GO analysis, including oxygen transport, gas transport, cellular response to toxic substance, specific granule lumen, cytoplasmic vesicle lumen, haptoglobin binding, hemoglobin binding, iron ion binding, etc. The key terms were selected for graphical display (Fig 5A). 7 significantly enriched pathways (S3 Table) were obtained from the KEGG analysis (Fig 5B). The specific signaling pathways included African trypanosomiasis, malaria, nitrogen metabolism, arginine biosynthesis, base excision repair, biosynthesis of amino acids, and sulfur metabolism.

### 3.5 Identification, diagnostic accuracy assessment, and PPI network construction of key genes

**Fig 6:**
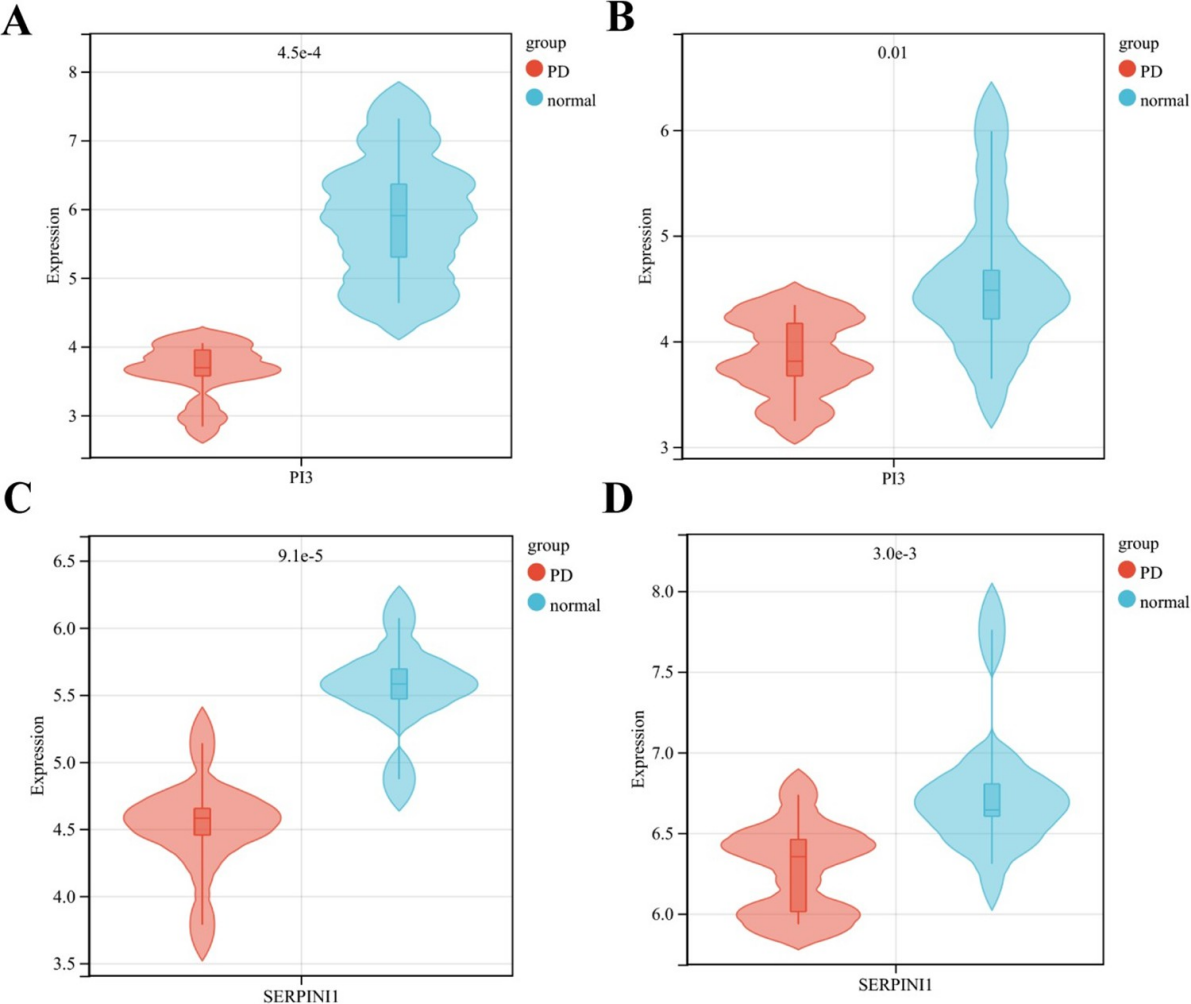
Identification and diagnostic accuracy assessment of key genes. The box plots of PI3 expression levels between PD and normal samples in the training dataset (A) and validation dataset (B), and SERPINI1 expression levels between PD and normal samples in the training dataset (C) and validation dataset (D).

6 significantly different genes, including SERPINI1, PI3, glutamate-ammonia ligase, late cornified envelope 1C, lipocalin 2, and interleukin 18 (S4 Table), were found between PD and normal samples in the validation dataset, of which 2 down-regulated genes, PI3 and SERPINI1, were consistent in the two datasets. The box plots were drawn for the expression levels of the two genes in the training and validation datasets (Fig 6A–6D).

**Fig 7:**
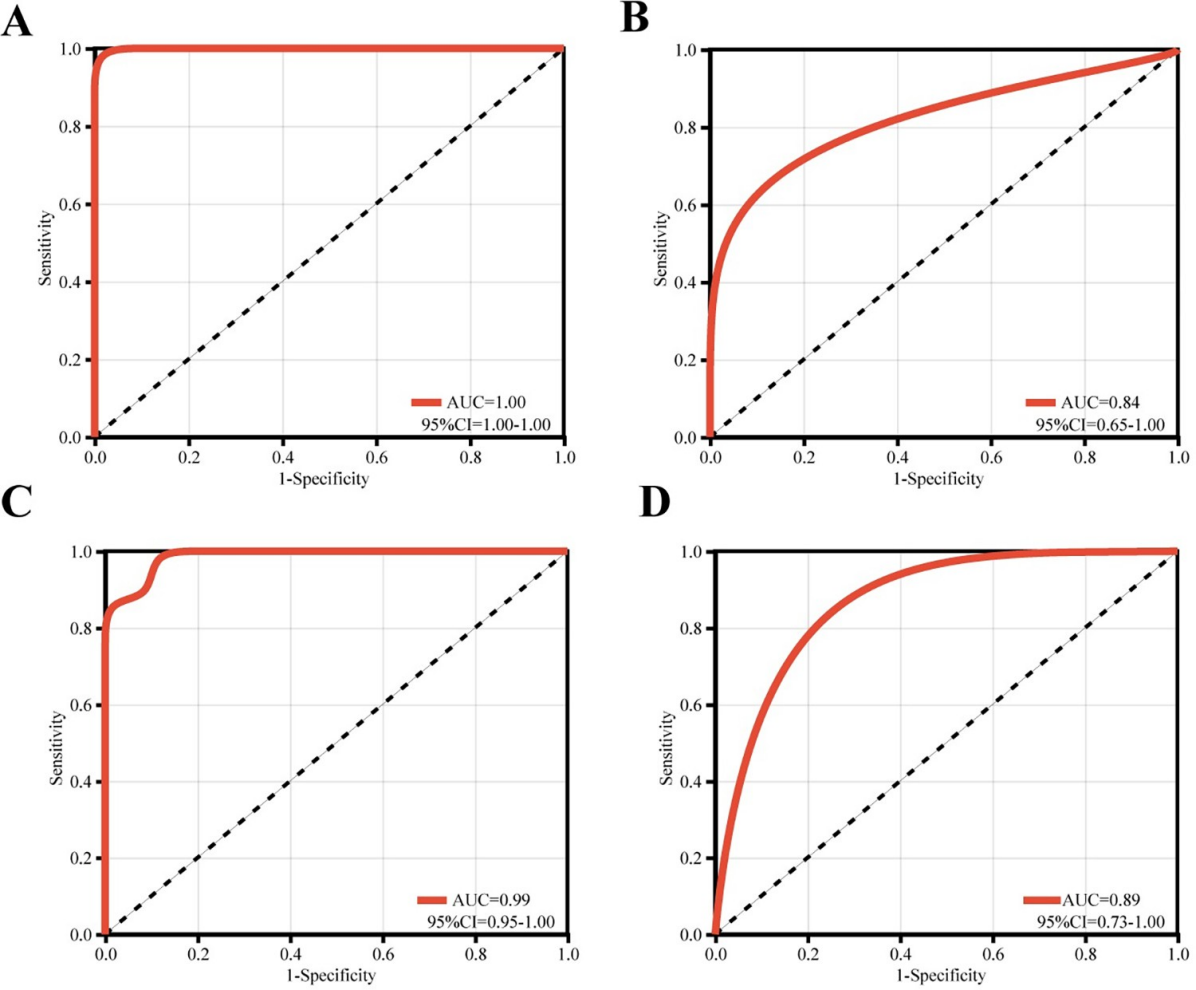
The ROC curves of PI3 in the training dataset (A) and validation dataset (B), and that of SERPINI1 in the training dataset (C) and validation dataset (D).

**Fig 8:**
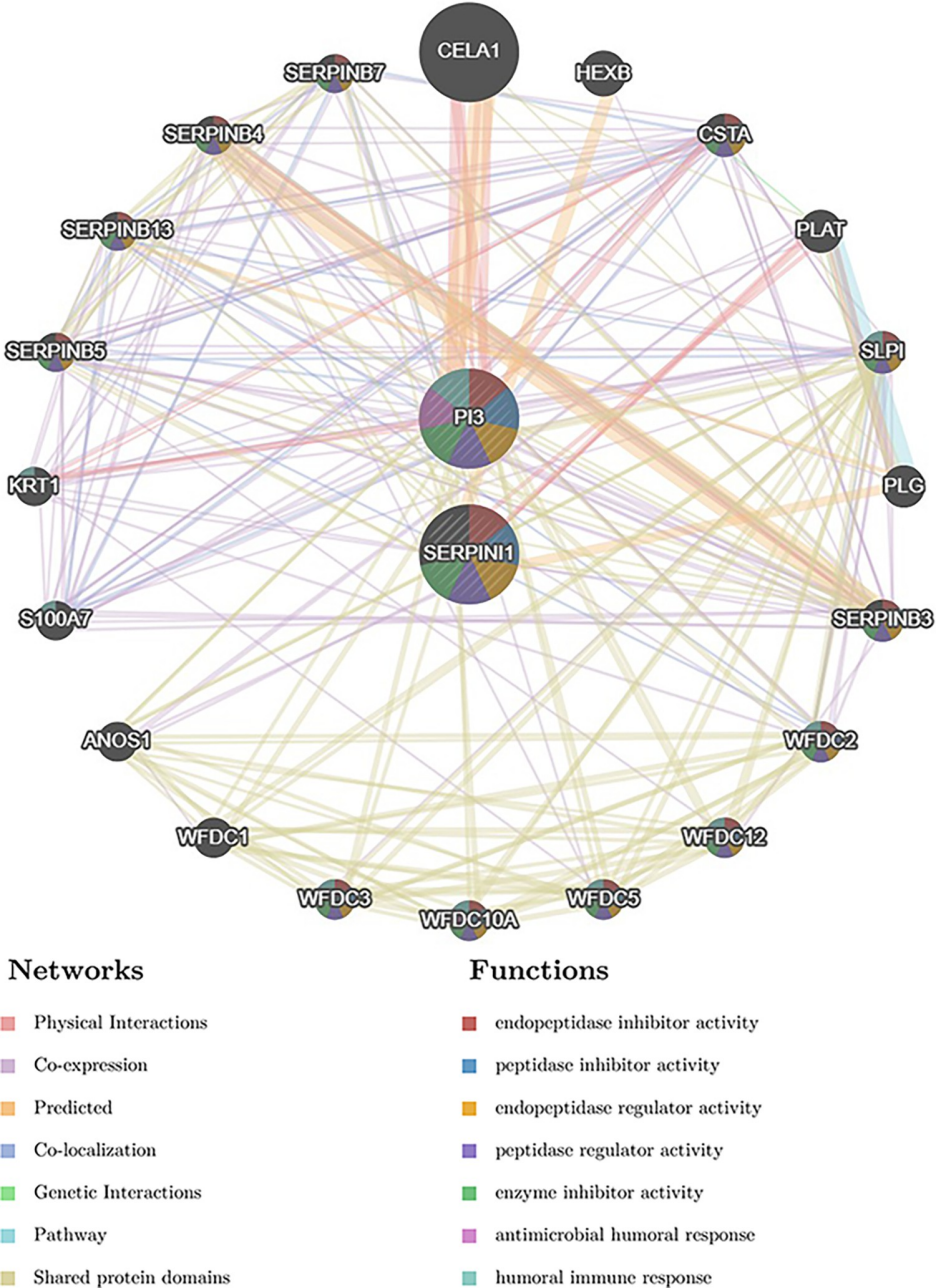
The PPI network of the key genes.

The ROC curves were plotted in all datasets of the key genes to assess the diagnostic accuracy through the pROC package in R language. It turned out that the AUC values for the key genes were greater than 0.7 in both datasets (Fig 7A–7D). What’s more, the PPI analysis was performed using the GeneMANIA database (Fig 8). The main significantly enrichment pathways included endopeptidase inhibitor activity, peptidase inhibitor activity, endopeptidase regulator activity, peptidase regulator activity, enzyme inhibitor activity, antimicrobial humoral response and humoral immune response, etc.

### 3.6 Key miRNAs screening and KEGG pathway enrichment analysis

**Fig 9:**
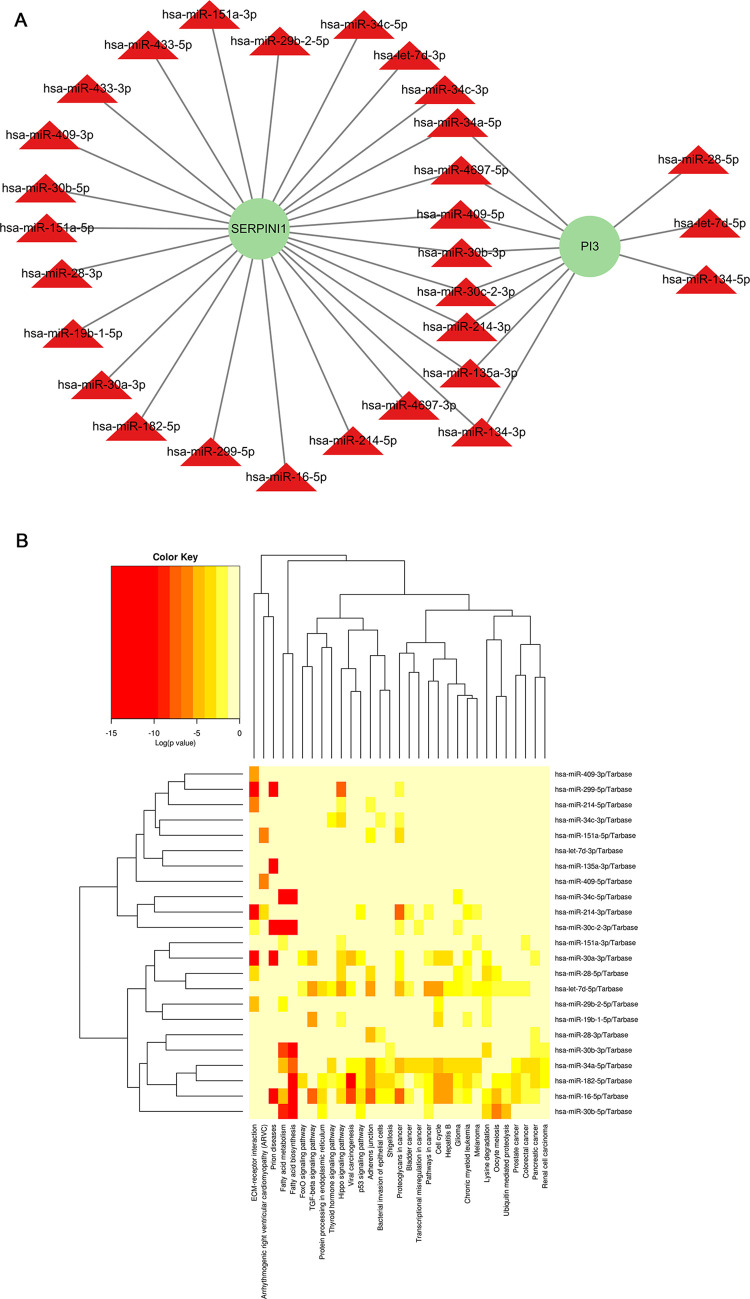
Screening and KEGG pathway enrichment analysis of key miRNAs. (A) The network of key genes and miRNAs (the red triangles were key miRNAs and the green circles were key genes). (B) The significantly enriched KEGG pathways of key miRNAs (red indicated more significantly enriched).

Based on the HMDD database, 131 miRNAs related to PD were obtained and intersected with the miRNAs associated with key genes. The miRNAs with a score of 1 were selected to obtain 29 key miRNAs like hsa-miR-30c-2-3p, hsa-miR-34a-5p, hsa-miR-4697-5p, etc. A network was constructed based on the key genes and miRNAs (Fig 9A), which were enriched by KEGG (Fig 9B). The main KEGG pathways encompassed cell cycle, proteoglycans in cancer, protein processing in endoplasmic reticulum, adherens junctions and chronic myeloid leukemia.

### 3.7 RT-qPCR confirmation

**Fig 10:**
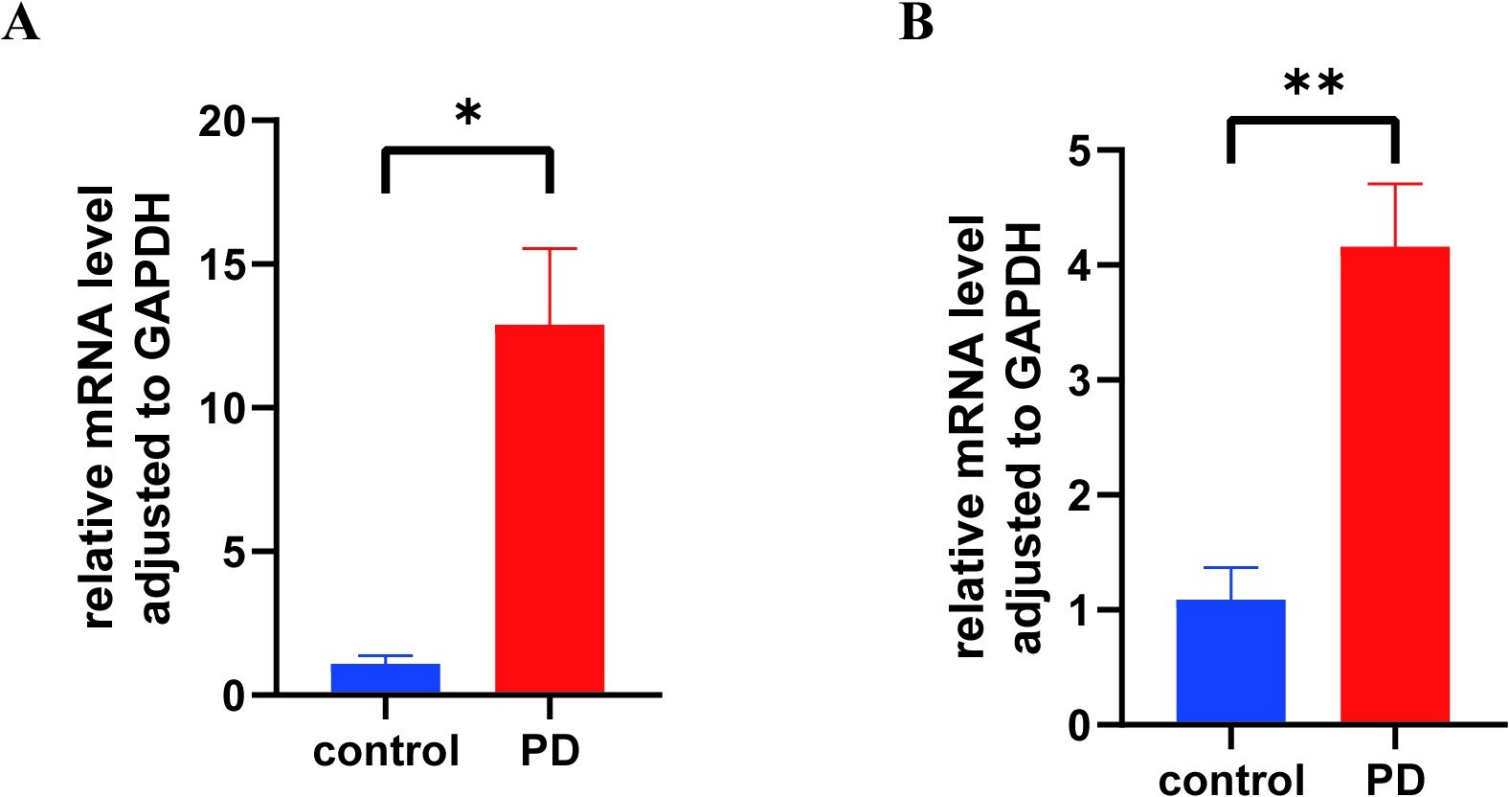
The RT-qPCR results of 2 hub genes. (A) The RT-qPCR results of PI3 between PD and the control group. (B) The RT-qPCR results of SERPINI1between PD and the control group. *: P<0.05; **: P<0.01.

The expression of 2 hub genes, PI3 and SERPINI1 were verified by the integration bioinformatics analysis. Based on the qRT-PCR results, the expressions of PI3 (*P* = 0.045, Fig 10A) and SERPINI1 (*P* = 0.008, Fig 10B) were up-regulated in PD compared to the control group, which were inconsistent with the results of the bioinformatics analysis.

## 4. Discussion

PD is a kind of disease that seriously endangers human health globally. Although there is rapid progress and evolution in technology to unravel the pathogenic factors of PD, the underlying pathogenesis remains to be determined. Copper is a transition metal linked to pathological and beneficial effects in neurodegenerative diseases [36], as both excess copper levels and copper deficiency can be harmful [11]. There is evidence that excess copper leads to neuronal cell death and α-synuclein aggregation [36, 37]. Accordingly, Cu^II^[atsm] has been demonstrated to rescue dopaminergic cell loss and improve motor dysfunction based on both sporadic (toxin-based) and genetic mouse models of PD [38]. A meta-analysis based on the 18 eligible studies identified that substantia nigra copper levels in PD patients were significantly lower than the control cases [39]. Consequently, the controversial association should be further studied to reveal the underlying mechanisms. Cuproptosis is a mode of cell death associated with lipoylated TCA enzymes in mitochondrial metabolism, different from apoptosis, ferroptosis, and necroptosis [14]. It has attracted tremendous interests in the field of tumor studies. However, it needs further investigation to determine the biological processes and signal pathways related to cuproptosis in PD.

In this study, we acquired PD and normal samples from the database as the training and validation datasets and compared the expression levels of CRGs and DEGs between PD and normal samples to obtain the differentially expressed CRGs. The disease module-related genes and the key differentially expressed CRGs were respectively obtained by WGCNA and PPI, and they were intersected to observe the intersection genes. Then we enriched the genes by the KEGG and GO analysis. Next, we identified 2 key genes from the datasets, PI3 and SERPINI1, and constructed a PPI network. They were verified by the ROC curves (AUC>0.7). Then the PD-related miRNAs and key genes-related miRNAs were intersected into key miRNAs, which were enriched by the KEGG analysis. At last, the results were verified by RT-qPCR. Technically, we integrated the bioinformatics analysis and the biological experiment to figure out not whether cuproptosis-related genes were involved in the pathogenesis of PD. To date, we utilized these methods for the first time to explore the association between cuproptosis and the disease, innovatively elucidating the underlying pathogenesis from a new perspective and laying the foundation for subsequent basic experiments. It’s worth mentioning that the intervention against the corresponding cuproptosis-related molecules would provide promising targets for PD therapy as well as diagnostic biomarkers deserving further investigation.

From the data analysis in this study, we obtained 2 key genes, PI3 and SERPINI1, respectively. PI3, which functions in preventing excessive tissue injury during inflammatory events [40], has a significant association with a diversity of tumors [41]. Besides, one recent study based on pathway clustering analysis found that PI3 is upregulated in PD patients [42], which was consistent with our experimental results. Therefore, how PI3 participates in PD patients could be investigated in the future to explain this contradictory finding, possibly related to the different stages of neuroinflammation. SERPINI1 encodes an axonally secreted neuroprotective protein called neuroserpin, which values in synaptic plasticity [43]. It is implicated in the conformational disease familial encephalopathy with inclusion bodies, which manifested as progressive dementia and epilepsy via endoplasmatic reticulum (ER)-overload response [44]. In a study based on analyzing human SERPIN transcripts, SERPINI1 was significantly downregulated in Sporadic Creutzfeldt-Jakob disease patients [45]. Besides, the role of SERPINI1 in AD pathology is controversial. Neuroserpin can act neuroprotective by binding to Aβ and altering its oligomerization [46], however, it could also be detrimental in reducing the clearance of Aβ [47]. The conflicting role in AD is similar to the gap between our bioinformatics predictions and experimental results, which requires further studies. What’s more, aggregated α-synuclein has been demonstrated to induce ER fragmentation and compromise ER protein folding capacity [48]. Therefore, the role of SERPINI1 could be further investigated in other neurodegenerative diseases like PD targeting α-synuclein and ER homeostasis. Chymotrypsin-like elastase family member 1 was predicted as a key interacting gene by PPI analysis of the key genes. It is a digestive protease expressed during lung development and regeneration [49]. However, its role in PD remains unclear and needs to be further clarified.

What’s more, the interaction prediction models targeting gene, mRNA, miRNA, lncRNA [50, 51] proteins [52], metabolites [53, 54] and even the drug compounds [55, 56] have developed in the fields of computational biology, which could provide fresh and valuable insights into the genetic markers related with the disease. In recent years, with the popularity of single-cell sequencing, the deep-learning methods for single-cell clustering analysis have also emerged consequentially with great prospects for development [52, 57]. Generally, the rapid growth of the computational biological methods results in a deep and comprehensive insight of the disease from the molecular level. Herein, by intersecting the related miRNAs of PD and key genes, we screened 29 key miRNAs including hsa-miR-30c-2-3p, hsa-miR-34c-5p, and hsa-miR-30b-5p, etc. Consistent with our results, another study found that the level of hsa-miR-30c-2-3p in plasma extracellular vesicles was significantly higher in PD patients than that in controls, and the target genes were enriched in dopaminergic synapse and PD pathway [58]. Eventually, we predicted fatty acid biosynthesis and fatty acid metabolism as the key pathways by KEGG. Considering the significance of lipoylated proteins in cuproptosis, it is reasonable to infer that cuproptosis takes part in the pathogenesis of PD.

Currently, there are few bioinformatic analyses targeting cuproptosis in PD as mentioned previously [18–20].A recent study was dedicated to exploring the potential CRGs, the immune infiltration patterns in PD, and consequently, the associated copper chelators therapy [18]. Another study showed that CRGs were significantly enriched in immunity-related pathways like JAK-STAT and Notch pathway. Based on the LASSO analysis, core genes were identified and utilized in a prognostic prediction model [19]. Recently, Zhang et al. found that Cu exposed to mice lead to neuronal degeneration and promoted the expression of cuproptosis-related proteins ferredoxin 1 and dihydrolipoamide S-acetyltransferase, providing experimental evidence for the involvement of cuproptosis in neurodegenerative diseases [59]. In contrast to the above articles, the systematic bioinformatic and experimental methods were simultaneously used in our study to screen and validate the hub genes. Above all, the identification of key genes and miRNAs is of guiding value for exploring the underlying pathogenesis, the biomarkers for early identification, and the potential candidates for drug targeting.

However, it remains some limitations in our study as listed. 1) the limited sample size we obtained from public databases may not only affect the accuracy of predicting the key genes but also restrict the subgroup analysis, so it could be further designed in a larger size of the samples. What’s more, the samples could be stratified by gender and other demographic characteristics, and PD could be categorized into different subtypes in future studies. 2) The algorithms in our study could be affected by the adoption of the coefficients and calculation methods, which could have impact on the results. Future research could integrate diverse computational biology algorithms and methods, such as ordinary differential equations based theoretical models on gene and protein signaling networks [60–62], to obtain the results robustly from different perspectives. 3) The results of the experimental validation are contradictory to our predictions. The complicated results might be related with the post-translation modification, the different stages of the disease, etc. Therefore, the in-depth studies could be conducted to figure out the underlying mechanisms.

## 5. Conclusion

In summary, our study demonstrates that cuproptosis contributes to the development of PD through comprehensive bioinformatics approaches combined with experimental verification. Future studies could be designed to figure out the pathogenic role of cuproptosis in PD based on larger populations and other algorithms like ordinary differential equations based theoretical models. The key genes and mi-RNAs obtained from the study could be instructive in the identification of the diagnostic biomarkers as well as the candidates for molecular modification and metal homeostatic therapies in the early course of the disease.

## Supporting information

S1 Table Cuproptosis-related genes. The Cuproptosis-related genes used in the manuscript.
(DOCX)

S2 Table The intersection genes of WGCNA and PPI. The intersection genes obtained from WGCNA and PPI.
(DOCX)

S3 Table Enriched KEGG pathways. The 7 pathways significantly enriched in the KEGG analysis.
(DOCX)

S4 Table The significantly different genes in the validation dataset. The significantly differentially expressed genes obtained in the validation dataset.
(DOCX)

## Abbreviations

AD: Alzheimer’s disease
AUC: Area Under Curve
BP: biological process
CC: cellular component
CRG: cuproptosis-related gene
DEG: differentially expressed gene
ER: endoplasmatic reticulum
FDR: False Discovery Rate
GO: Gene Ontology
KEGG: Kyoto Encyclopedia of Genes and Genomes
MF: molecular function
PD: Parkinson’s disease
PI3: peptidase inhibitor 3
PPI: protein-protein interaction
ROC: receiver operating characteristic
SERPINI1: neuroserpin family I member 1
STRING: Search Tool for the Retrieval of Interacting Genes
TCA: tricarboxylic acid
WGCNA: weighted gene co-expression network analysis
