# Supplementary material for: Identification of cuproptosis-realated key genes and pathways in Parkinson’s disease via bioinformatics analysis
Source: PLoS One. 2024 Apr 16;19(4):e0299898. doi: 10.1371/journal.pone.0299898 (PMC11020840; doi:10.1371/journal.pone.0299898)
Supplement: S1 Table — The Cuproptosis-related genes used in the manuscript. (DOCX) [file pone.0299898.s001.docx]

**S1 Table.** Cuproptosis-related genes.

| **Gene Symbol** | **Gene Name** |
| --- | --- |
| ATP7A | ATPase copper transporting alpha |
| ATP7B | ATPase copper transporting beta |
| CDKN2A | Cyclin dependent kinase inhibitor 2A |
| DBT | Dihydrolipoamide branched chain transacylase E2 |
| DLAT | Dihydrolipoamide S-acetyltransferase |
| DLD | Dihydrolipoamide dehydrogenase |
| DLST | Dihydrolipoamide S-succinyltransferase |
| FDX1 | Ferredoxin 1 |
| GCSH | Glycine cleavage system protein H |
| GLS | Glutaminase |
| LIAS | Lipoic acid synthetase |
| LIPT1 | Lipoyltransferase 1 |
| LIPT2 | Lipoyl(octanoyl) transferase 2 |
| MTF1 | Metal regulatory transcription factor 1 |
| NFE2L2 | NFE2 like bZIP transcription factor 2 |
| NLRP3 | NLR family pyrin domain containing 3 |
| PDHA1 | Pyruvate dehydrogenase E1 subunit alpha 1 |
| PDHB | Pyruvate dehydrogenase E1 subunit beta |
| SLC31A1 | Solute carrier family 31 member 1 |
