# Supplementary material for: Identification of cuproptosis-realated key genes and pathways in Parkinson’s disease via bioinformatics analysis
Source: PLoS One. 2024 Apr 16;19(4):e0299898. doi: 10.1371/journal.pone.0299898 (PMC11020840; doi:10.1371/journal.pone.0299898)
Supplement: S2 Table — The intersection genes obtained from WGCNA and PPI. (DOCX) [file pone.0299898.s002.docx]

**S2 Table.** The intersection genes of WGCNA and PPI.

| **No.** | **Gene** | **Module** |
| --- | --- | --- |
| 1 | LCE1C | black |
| 2 | LCE1D | black |
| 3 | NEUROG3 | black |
| 4 | SF3A2 | black |
| 5 | SOX17 | black |
| 6 | THY1 | black |
| 7 | SLC4A1 | turquoise |
| 8 | AHSP | turquoise |
| 9 | HBD | turquoise |
| 10 | EPB42 | turquoise |
| 11 | LCN2 | turquoise |
| 12 | ELANE | turquoise |
| 13 | CA1 | turquoise |
| 14 | HBM | turquoise |
| 15 | HBA2 | turquoise |
| 16 | HBQ1 | turquoise |
| 17 | HBA1 | turquoise |
| 18 | GYPB | turquoise |
| 19 | SELENBP1 | turquoise |
| 20 | PI3 | turquoise |
| 21 | SKA3 | turquoise |
| 22 | BPI | turquoise |
| 23 | DEFA4 | turquoise |
| 24 | IL18 | turquoise |
| 25 | CKS2 | turquoise |
| 26 | CENPW | turquoise |
| 27 | ARG1 | turquoise |
| 28 | ZNF277 | turquoise |
| 29 | POLB | turquoise |
| 30 | POLE2 | turquoise |
| 31 | FAS | turquoise |
| 32 | SERPINI1 | turquoise |
| 33 | SLC25A39 | turquoise |
| 34 | CCAR1 | turquoise |
| 35 | GLUL | turquoise |
| 36 | TCN1 | turquoise |
