# Supplementary material for: Identification of cuproptosis-realated key genes and pathways in Parkinson’s disease via bioinformatics analysis
Source: PLoS One. 2024 Apr 16;19(4):e0299898. doi: 10.1371/journal.pone.0299898 (PMC11020840; doi:10.1371/journal.pone.0299898)
Supplement: S3 Table — The 7 pathways significantly enriched in the KEGG analysis. (DOCX) [file pone.0299898.s003.docx]

**S3 Table.** Enriched KEGG pathways.

| **KEGG Pathway** | **Count** | ***P.* VALUE** | **Gene** |
| --- | --- | --- | --- |
| African trypanosomiasis | 4 | 1.8572e-06 | HBA2, HBA1, IL18, FAS |
| Malaria | 4 | 5.8433e-06 | HBA2, HBA1, GYPB, IL18 |
| Nitrogen metabolism | 2 | 0.0008 | CA1, GLUL |
| Arginine biosynthesis | 2 | 0.0012 | ARG1, GLUL |
| Base excision repair | 2 | 0.0031 | POLB, POLE2 |
| Biosynthesis of amino acids | 2 | 0.0151 | ARG1, GLUL |
| Sulfur metabolism | 1 | 0.0250 | SELENBP1 |
