# Supplementary material for: Identification of cuproptosis-realated key genes and pathways in Parkinson’s disease via bioinformatics analysis
Source: PLoS One. 2024 Apr 16;19(4):e0299898. doi: 10.1371/journal.pone.0299898 (PMC11020840; doi:10.1371/journal.pone.0299898)
Supplement: S4 Table — The significantly differentially expressed genes obtained in the validation dataset. (DOCX) [file pone.0299898.s004.docx]

**S4 Table.** The significantly different genes in the validation dataset.

| **Gene Symbol** | **Gene Name** | ***P.* VALUE** |
| --- | --- | --- |
| SERPINI1 | Serpin family I member 1 | 3.00e-03 |
| PI3 | Peptidase inhibitor 3 | 0.01 |
| GLUL | Glutamate-ammonia ligase | 0.01 |
| LCE1C | Late cornified envelope 1C | 0.02 |
| LCN2 | Lipocalin 2 | 0.02 |
| IL18 | Interleukin 18 | 0.03 |
